# Molecular detection and species identification of *Plasmodium* spp. infection in adults in the Democratic Republic of Congo: A population-based study

**DOI:** 10.1371/journal.pone.0242713

**Published:** 2020-11-23

**Authors:** Kahindo Kiyonga Aimeé, Thierry Bobanga Lengu, Célestin Ndosimao Nsibu, Solange Efundu Umesumbu, Dieudonné Mumba Ngoyi, Tie Chen

**Affiliations:** 1 Department of Clinical Immunology, Tongji Hospital, Tongji Medical College, Huazhong University of Sciences and Technology, Wuhan, People’s Republic of China; 2 Department of Tropical Medicine Infectious and Parasitic Diseases, University of Kinshasa, Kinshasa, Democratic Republic of Congo; 3 Institut National de Recherche Biomédicale (INRB), Kinshasa, Democratic Republic of Congo; 4 Department of Pediatrics, University Hospital of Kinshasa, Faculty of Medicine, University of Kinshasa, Kinshasa, Democratic Republic of Congo; 5 Programme National de Lutte Contre le Paludisme (PNLP), Kinshasa, République Démocratique du Congo; Instituto Rene Rachou, BRAZIL

## Abstract

**Background:**

In efforts to control malaria infection, the Democratic Republic of Congo has implemented several strategies. Studies assessing their efficiency mainly involved at-risk groups, especially children under five years of age. This study aimed to determine the prevalence and identify the risk factors associated with *Plasmodium* spp. infection.

**Methods:**

From October 2014 to March 2015, individuals aged at least 15 years were selected randomly and enrolled in a cross-sectional study conducted throughout the country. Microscopy and polymerase chain reaction (PCR) analysis were used for the detection of *Plasmodium* ssp.

**Results:**

From 2286 individuals recruited, 1870 with valid laboratory results were included in the study for further analysis. The prevalence of *Plasmodium* spp. infection assessed by microscopy (355/ 1870 (19%) was lower than that estimated by PCR (580/1870 (31%). In addition, the difference between the two results was statistically significant (*P* < 0.0001). The most prevalent *Plasmodium* species was *P*. *falciparum*, either as mono-infection (96.3%; 95% C.I. 93.9–98.1) or combined with *P*. *malariae* (3.7%; 95% C.I. 2.8–5.9). The mean parasite density was 3272739 trophozoites/μL of blood. Women had higher risks of being infected than men (OR 2.03, 95% C.I.: 1.96. 2.62, *P* = 0.041)].

**Conclusion:**

In this study, the molecular detection and species identification of *Plasmodium* spp. showed that, despite all efforts for malaria control, malaria remains a public health problem in the Democratic Republic of Congo. The high prevalence and parasite density of *Plasmodium* spp. in adults make this age group a potential parasitic infectious reservoir for the at-risk groups and supports the need to include this age group in further programs for malaria control.

## 1. Introduction

*Plasmodium* spp. are pathogens transmitted through the bite of infected female anophelines, causing malaria. They are a genus of protozoan (single-celled) parasites that belong to the *Plasmodiidae* family. Four different species of *Plasmodium* have been identified in humans: *P*. *falciparum*, *P*. *malariae*, *P*. *ovale*, and *P*. *vivax*. Occasionally, humans become infected with *Plasmodium* species that generally infect animals, such as *P*. *knowlesi* [1]. Risk factors that have significant impacts on *Plasmodium* spp. transmissions include population immunity, social and economic status (housing conditions), application of mosquito control measures, and environmental factors [2]. Prevalence and risk factors associated with *Plasmodium* infection are used as indicators guiding public health interventions to track the progress of malaria control programs.

Malaria is a life-threatening parasitic disease, prevalent in many countries in Africa, south of the Sahara Desert. The Democratic Republic of Congo (DRC) is the second-leading country in the world in terms of malaria cases, after Nigeria, accounting for 11% of the 219 million malaria cases (including 435 000 deaths) in 2017 [3, 4]. Using data from the 2007 DHS, a study involving adults, conducted in the DRC, identified several risk factors for malaria, including age, gender, and individual and community-level wealth [5]. To reduce the malaria burden and in accordance with the WHO recommendations, the DRC implemented strategic plans for malaria control in 2007–2011, then 2011–2015, covering several areas of intervention, including prompt and effective handling of cases, promotion of individual and collective protective measures, use of long-lasting insecticidal mosquito nets (LLINs), intermittent preventive treatment and intra-home spraying, epidemiology, and fight against epidemics [6]. However, despite recent improvements in the coverage of malaria interventions, malaria remains a significant cause of morbidity and mortality in Africa, and the DRC in particular [6–8].

Previous studies have shown that people who are at high risk of mortality due to malaria are pregnant women, children under five years of age, immune-compromised patients, and travelers from non-endemic areas into malaria-endemic regions [9–12]. Therefore, various studies assessing the efficiency of different strategies for malaria control in the DRC mostly focused on these at-risk groups, especially children under five years of age [13–16]. Adults were reported to receive more mosquito bites and thereby constitute a potential infectious reservoir [17]. However, this age group did not attract similar attention as the at-risk groups. Therefore, this study aimed to investigate the carriage of *Plasmodium* spp., specifically in adults in the DRC. We determined the prevalence, identified the parasite species, assessed the parasite density in infected subjects, and identified the risk factors associated with *Plasmodium* spp. infection in adults.

## 2. Materials and methods

### 2.1 Study design, sites, and participants

This was an observational population-based cross-sectional study conducted from October 2014 to March 2015 in all the provinces of the DRC (before the current territorial division) in the 13 functional sentinel sites of the National Program for the Fight against Malaria (Programme National de Lutte contre le Paludisme, PNLP).

The sample size was calculated using a standard formula for prevalence studies [18] as follows:

$$n=\frac{{ZP}^{2}\;(1-P)}{d^{2}}$$

where *n* is the sample size, *and Z* is a *Z* statistic value of 1.96 at a confidence level of 95%. *P* is considered prevalent at 33.5% (prevalence of malaria in adults aged 15–59 years / DHS-DRC 2007 in DRC) [19] at 95% confidence interval, and *d* is a 5% relative precision. To account for dropout and missing samples during the study, 20% of the calculated sample size was added. Therefore, a targeted sample size of 410 was obtained to assess the prevalence of *Plasmodium* spp. in adults in the DRC.

A probabilistic sampling with four levels was carried out as sampling technique (**S1 Fig**). First, we randomly selected three health areas that accounted for each sentinel site (first level). Then, we randomly selected ten villages or streets by health area (second level). At the third level, we first proceeded to the plots observed in the streets or villages selected the day before, under the guidance of the supervisor. Then, by systematic sampling, we selected six households per street or village after determining the sampling interval, starting with the households that were randomly selected. Finally, in plots with several households, we carried out a simple randomized sampling of the household to be surveyed (fourth level).

To be included in the study, the subject should be a member of the selected household, agree to participate in the study by signing a written informed consent form, and have at least 15 years of age, with or without malaria symptoms. In this study, we defined an adult as a respondent of at least 15 years of age. We chose to study this group as a homogeneous group because it is usually not considered as the main target of most programs for malaria prevention and eradication.

This study was reviewed and approved by the ethical committees of the School of Public Health, Kinshasa University, DRC. Before inclusion, written informed consent was obtained from each participant/ legal guardian for minors. All measures were taken to avoid contamination during sampling (the standards of good laboratory practice were respected), and the results obtained were confidential. Malaria-positive cases were treated with antimalarial drugs based on the current national treatment guidelines of the DRC.

### 2.2 Data collection

#### 2.2.1 Survey questionnaire

For the epidemiological survey, a questionnaire was sent to the household head or his representative. At the same time, samples were taken from members living under the same roof and meeting the inclusion criteria. The following sociodemographic, housing, and economic variables were recorded: study site (province, health zone, and health area), age, gender, possession of an LLIN, number of LLIN, schooling, level of education, marital status, number of persons in the household, number of rooms, and number of beds per household.

#### 2.2.2 *Plasmodium* spp. microscopical diagnosis

For each selected participant, blood samples were collected from a finger prick. Thick and thin blood smears were prepared, dried, and stained with Giemsa 10% for 10 min. Thin smears were fixed with methanol before staining. Blood slides were examined by light microscopy at 1,000 × magnification.

The parasite density was calculated by counting the number of asexual parasites per 200 leukocytes in the thick blood film. A laboratory technician counted 500 leukocytes before considering a slide as negative. When thick films were positive, thin films were read for species determination. Based on an assumed 8000 white blood cells/μL [20], the PD/μl was calculated using the following formula:

$$Parasites/\mu l\;blood=\frac{Number\;of\;parasites\;counted\;x\;8000\;white\;cells/\mu L}{No.of\;white\;cells\;counted}$$

All slides were read twice by experienced microscopists. One was from the Parasitology Department of the University of Kinshasa, and the other from the National Malaria Reference Laboratory at the National Institute for Biomedical Research (INRB). If the discrepancy was greater than 15%, a third reader was used to confirm the diagnosis.

#### 2.2.3 DNA extraction and molecular analysis

Blood samples were obtained from a finger prick and dropped on filter paper (Whatman 3MM®), dried and stored in individual plastic bags with desiccant. *Plasmodium* DNA was extracted using the QIAamp DNA Mini Kit® (Qiagen Benelux, Venlo, Netherlands), according to the manufacturer’s recommendations. Briefly, two circles of approximately 3 mm diameter were punched out from a blood spot and placed into a 1.5 mL microcentrifuge tube in which 180 μL of buffer ATL was added. The final elution volume was 150 μL. Each dried blood spot was treated individually in a sterile petri dish to avoid contamination. One negative control (sterile water) was included for each dozen of blood samples.

#### 2.2.4 Molecular detection of *Plasmodium* spp. Infection

Parasite identification was based on PCR assay targeting the 18S rRNA gene. The 18S rRNA gene was used as a target since it contains both highly conserved and variable regions for each *Plasmodium* species. At least five copies of the gene are dispersed on separate chromosomes of *Plasmodium* [21]. Real-time PCR, having a sensitivity varying from 0.02 to 0.006 parasites/μL spots treated. DNA was stored at −20°C until further analysis. Human *Plasmodium* species identification was run, as previously described by CNOPS *et al*. [22]. PCR tests were run on a light cycler 480 instrument (Roche®) and in the presence of positive control. PCR conditions were as follows: 2 min at 95°C, followed by 50 cycles of 15 s at 95°C and 60 s at 60°C. Submicroscopic malaria infection was defined as malaria infection identified by PCR from individuals with malaria slides negative by microscopy.

### 2.3 Statistical analysis

Data were double-entered and validated in Microsoft Excel 2007 software and analyzed using Epi-Info version 3.5.3 software. The quantitative variables were summarized as mean ± standard deviation (SD). The median and interquartile range (IQR) were used when the dispersion of values apart from the SD was relatively large. The categorical variables were summarized as frequencies and compared using the Pearson chi-square test. The odds ratios (ORs) with 95% confidence intervals (CIs) were used to measure the association *between P*. *ssp* carriage and study variables. A P-value of less than 0.05 was considered statistically significant.

## 3. Results

Our analysis included 1870 adults out of the 2286 initially selected for inclusion in this study. For 416 individuals, laboratory results were not taken into account due to several reasons, including disagreement for blood sampling by the subject or slides poorly stained with Giemsa (**S2 Fig**). Data from the Equateur province were missing during analysis.

### 3.1 Characteristics of the study population

#### 3.1.1 Age and distribution of respondents by gender

The median age of our study population was 36 years (IQ: 28.5–47), with extremes of 15 and 93 years. The study population comprised more female (60.6%) than male respondents.

#### 3.1.2. Distribution of respondents by province, health zones and health areas

The distribution of the study population by province, health zones, and health area is shown in **Table 1.**

**Table 1:** Distribution of respondents by health zones and health areas.

Provinces	Health zones	N (%)	health areas	N (%)
**Bandundu**	Vanga		Terre jaune	61(2.70)
		181(7.90)	Zaba lunuingu	60 (2.60)
			Zaba kilundu	60(2.60)
**Bas-Congo**	Kimpese		Kimbala	57 (2.50)
		177(7.70)	Kilueka	60(2.60)
			Malanga	60(2.60)
**Kasai Occidental**	Mikalayi		Mikalayi	61(2.70)
		181(7.90)	Kabue	60(2.60)
			Matamba	60(2.60)
**Kasai Oriental**	Mwene ditu		Matobo	61(2.70)
		179(7.80)	Musadi	58(2.50)
			Bondoyi	60(2.60)
**Katanga**	Fungurume		Mpala	59(2.60)
		180(7.90)	Dipetai	60(2.60)
			Kilusonsa	61(2.70)
	Kapolowe		Kibangu	60(2.60)
		180(7.90)	Lupidi	60(2.60)
			Kapolowe station	60(2.60)
**Kinshasa**	Kingasani		Nsanga	60(2.60)
		182(8.00)	Lisanga	62(2.70)
			Atandele	60(2.60)
			Pinzi	64(2.80
	Kalamu 2	189(8.30)	Yolo sud 1	65(2.80)
			Yolo-nord 3	60(2.60)
**Maniema**	Salamabila		Salamabila	60(2.60)
		180(7.90)	Kimbaseke 1	60(2.60)
			Camp central	60(2.60)
	Kalima		Kakutya ii	60(2.60)
		177(7.70)	Kakaleka	58(2.50)
			Kinkungwa	59(2.60)
**Nord Kivu**	Musienene		Ngoma	59(2.60)
		122(5.30)	Ivatama	**63**(2.80)
**Province Orientale**	Kabongo		Focus	60(2.60)
		178(7.80)	Umoja	59(2.60)
			Yabiso	59(2.60)
**Sud Kivu**	Katana		Ciranga	60(2.60)
		180(7.90)	Kabushwa	60(2.60)
			Luhihi	60(2.60)
**Total**		**2286 (100)**		**2286 (100)**

The majority of respondents came from the provinces of Kinshasa (16.20%), Katanga (15.70%), and Maniema (15.60% health areas) (**Table 1**).

### 3.2 Prevalence of *Plasmodium* spp. infections in the DRC

#### 3.2.1 Prevalence of *Plasmodium* spp. infections in DRC

**Fig 1:**
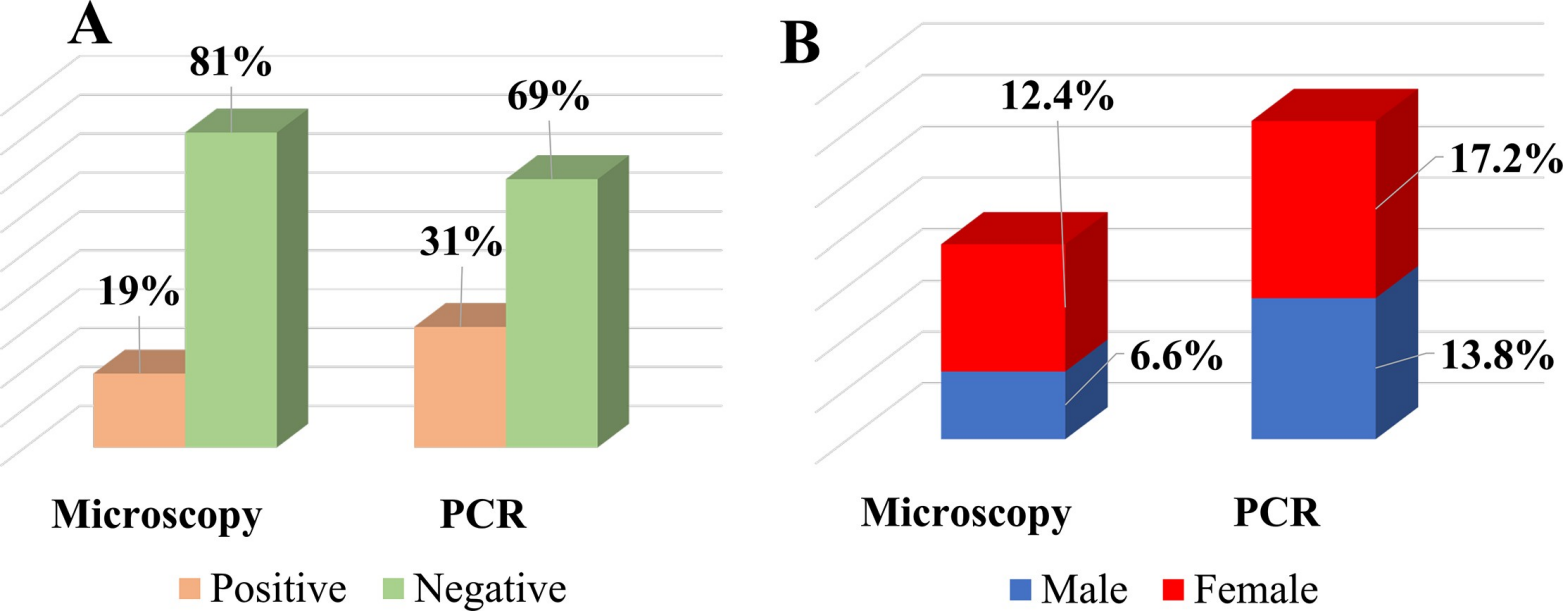
Prevalence of *Plasmodium* ssp infection using microscopy and PCR analysis. **A.** Comparison of the prevalence obtained by microscopy and PCR analysis. **B.** Repartition of *Plasmodium spp* infection by sex. Significance level **** = *P* < 0.0001.

The overall prevalence of *Plasmodium* spp. infections among adult Congolese and the reparation of infected patients by sex are shown in Fig 1.

The prevalence of *Plasmodium* spp. infections determined by PCR was significantly higher than that obtained using microscopy analysis, being 580/1870 (31%) and 355/1870 (19%) (*P* < 0.0001), respectively (**Fig 1**). Additionally, the prevalence of submicroscopic infection was 42.2%.

In addition, we found that women were more likely to be affected than men, with a prevalence of 232/1870 (12.4%) and 322/1870 (17.2%) by microscopy and PCR%, respectively. For men, the prevalence was 123/1870 (6.6%) and 258/1870 (13.8%) by microscopy and PCR%, respectively (**Fig 1, S1 Table**).

#### 3.2.2 Prevalence of *Plasmodium* infections by province

**Fig 2:**
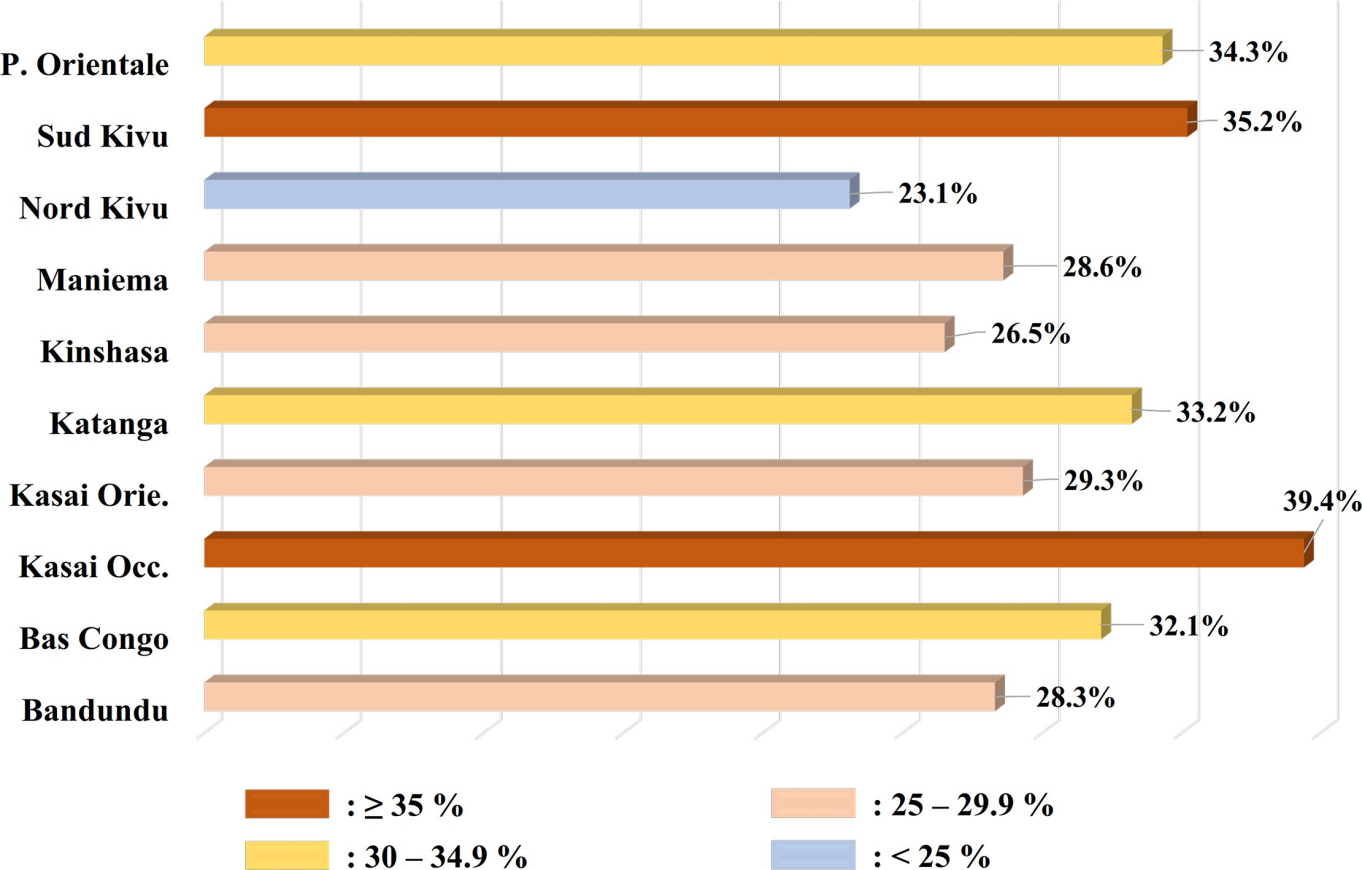
Prevalence of *Plasmodium* ssp infection by province using PCR analysis.

The prevalence of *Plasmodium* spp. infection by province, using PCR analysis is shown in **Fig 2**.

There was a disparity in the prevalence distribution across provinces. The results from both microscopy (**S2 Table**) and PCR analysis (**Fig 2, S3 Table**) revealed that the highest prevalence was observed in Kasai Occidental, with 45/147 (30, 6%) and 58/147 (39.4%) by microscopy and PCR, respectively; followed by the provinces of Sud Kivu and Province Orientale.

#### 3.2.3 Plasmodium species

Microscopy analysis revealed the presence of P. falciparum on all positive slides to thin smears. Nevertheless, the results of PCR analysis showed that P. falciparum was the most prevalent species, either as mono-infection (96.3%; 95% CI 93.9–98.1) or coinfection with P. malariae (3.7%; 95% C.I. 2.8–5.9). The other human Plasmodium species were not found.

### 3.3 Parasite densities

**Table 2** presents the parasite density in subjects with *Plasmodium* spp. infection.

**Table 2:** Distribution by parasite density.

	Parasite density (trophozoites / μl)		
	Mean ± SD	Median	Min-Max
DRC (National level)	3272, 73 ± 14165, 26	248	5–196631
**PROVINCES**			
Bandundu	5111.28 ± 14177, 58	280	7–64400
Bas Congo	319.44 ± 261.53	261	932–92
Kasai Occidental	**6106.04 ± 29044.49**	209	5–196631
Kasai Oriental	631.52 ± 1128.30	761	32–51180
Katanga	2654 ± 6177.27	320	12–36869
Kinshasa	**8202 ± 3114.53**	120	12–132800
Maniema	1766, 77 ± 3981.74	369	16–21600
Nord Kivu	5993.81 ± 14839, 96	243	11–45000
Sud Kivu	2404.96 ± 6836.02	208	5–36869
Province Orientale	1815.55 ± 3576.68	354	32–15307

The mean parasite density was 3272739 ± 14165, and 2678 trophozoites/μl of blood. Given the large dispersion around the mean, we calculated the median and found 248 trophozoites/μL of blood, with a minimum of 5 and a maximum of 196 631 trophozoites/μl of blood in Kasai Occident Q1-3 (96–151) (**Table 2**). The highest mean parasite densities were found in the provinces of Kinshasa (8202 ± 3114.53 trophozoites/μl of blood) and Kasai Occidental (6106.04 ± 29044.49 trophozoites/μL of blood) (**Table 2**).

### 3.4 Possession of per household and use of LLIN by the population study

**Table 3** shows possession of at least one LLIN per household and the use of LLIN by the general population.

**Table 3:** Possession per household and use of LLIN.

	Coverage	Percentage
**DRC**	Possession of at least one LLIN per household	68.0%
**(National level)**	Use of LLIN by general population	48.3%
**PROVINCES**		
**Bandundu**	Possession of at least one LLIN per household	86,9%
	Use of LLIN by general population	71,2%
**Bas Congo**	Possession of at least one LLIN per household	75,6%
	Use of LLIN by general population	60,2%
**Kasai Occidental**	Possession of at least one LLIN per household	**55,3%**
	Use of LLIN by general population	**39,1%**
**Kasai Oriental**	Possession of at least one LLIN per household	66,2%
	Use of LLIN by general population	42,8%
**Katanga**	Possession of at least one LLIN per household	83,4%
	Use of LLIN by general population	54,7%
**Kinshasa**	Possession of at least one LLIN per household	60,3%
	Use of LLIN by general population	42,6%
**Maniema**	Possession of at least one LLIN per household	63.8%
	Use of LLIN by general population	44,3%
**Nord Kivu**	Possession of at least one LLIN per household	68,6%
	Use of LLIN by general population	47,0%
**Sud Kivu**	Possession of at least one LLIN per household	61,7%
	Use of LLIN by general population	39,6%
**Province Orientale**	Possession of at least one LLIN per household	58,4%
	Use of LLIN by general population	41,4%

We found that 68% of our study population possessed at least one LLIN per household, and 48.3% effectively used these LLIN. The lowest prevalence of possession per household and use of LLIN were observed in the provinces of Kasai Occidental, with 55.3% and 39.1%, respectively, followed by the Province Orientale, Kinshasa, and Sud Kivu (**Table 3**).

### 3.5 Relationship between *Plasmodium* spp. infection and various factors investigated in this study

**Table 4** shows the relationship between *Plasmodium* spp. infection and various factors investigated in this study.

**Table 4:** Relationship between *Plasmodium spp* infection and various socio-demographic factors, using bivariate analysis (unadjusted odds ratios).

Factors	Unadjusted OR	[95% CI]	*P value*
Gender			
• **Female**	**2.33**	**[1.73 - 2.81]**	**0.002**
School attendance			
• No	0.97	[0.65–1.35]	0.84
Study level			
• Primary school	1	[0.80–1.28]	0.90
Marital status			
• Single	1.34	[0.99–1.83]	0.06
Number of persons per household			
• ≥ 5	1.16	[0.85–1.59]	0.33
Number of rooms in the household			
• < 2	1.34	[0.86–2.08]	0.18
Number of beds			
• < 2	1.5	[0.89–2.62]	0.12
Possession of LLINs in the household			
• No	0.89	[0.67–1.19]	1.19
Using LLINs			
• No	0.99	[0.77–1.29]	1.29

OR odds ratio, CI confidence interval, LLINs: long-lasting insecticidal mosquito nets

Univariate analysis showed that women had a 2-fold increased risk of having *Plasmodium* spp. infection compared to men (OR 2.33, 95% C.I.: 1.73. 2.81), *P* = 0.02] (**Table 4**).

The relationship between *Plasmodium* spp. infection and sex, adjusted for the other sociodemographic variables using logistic regression analysis (adjusted ORs) is presented in **Table 5.**

**Table 5:** Relationship between *Plasmodium spp* infection and gender, adjusted for the other sociodemographic variables using logistic regression analysis (adjusted odds ratios).

Factors	Adjusted OR	[95% CI]	*P value*
Gender			
• **Female**	**2.03**	**[1.96 - 2.62]**	**0.041**
• Male	0.99	[0.77–1.29]	1.29

OR odds ratio, CI confidence interval.

Adjusted for gender, number of persons in the household, number of rooms, number of beds per household.

In the adjusted analysis (**Table 5**), the risk of *Plasmodium* spp. infection was 2-fold higher in females than in males (OR 2.03, 95% C.I.: 1.96. 2.62), *P* = 0.041].

## 4. Discussion

The present study aimed to determine the prevalence of *Plasmodium* spp. infection in Congolese adults, to identify *Plasmodium* species, assess parasite density, and identify the risk factors associated with *Plasmodium* infection in Congolese adults.

The overall prevalence of *Plasmodium* spp. infections in adults diagnosed by microscopy was 19% (CI: 17.2. 20.9). *Plasmodium falciparum* was identified in all the samples (100%) tested positive by thick smear (microscopy). The prevalence rate found in our study was similar to that reported in Kenyan adults (22%), also using microscopy [23]. However, our results were lower than the prevalence reported by DHS-DRC II (23%) in children aged 6–59 months in the DRC [15]. This difference may be due to the fact that, unlike adults living in an endemic area, children below 5 years of age have not yet acquired effective immunity against *P*. *falciparum* and constitute a risk group for severe malaria [24].

Consistent with previous studies using molecular analysis for *Plasmodium* detection in DRC [19, 25, 26], we observed that the prevalence of *Plasmodium* spp. detected by PCR (31%) was significantly higher than that detected by microscopical assays (19%), with a large proportion of submicroscopic infection (42.2%). In addition, we identified *P*. *falciparum* as the most prevalent *Plasmodium* species infecting humans. The use of PCR for parasite detection in this study offers substantial advantages compared with the microscopical method. The sensitivity of molecular assays for malaria is substantially higher than that of microscopy. In addition, it allows for better identification of parasite species.

**Table 6** summarizes the results of prior studies assessing *Plasmodium* spp. by PCR in DRC.

**Table 6:** Assessment of Plasmodium infection by PCR analysis in DRC.

Years	Authors	Settings	PCR	Plasmodium species (PCR)
2011	Taylor S.M. et al	Adults aged 15–59 y / DHS-DRC 2007	33.5%	*- P*. *falciparum* (90.4%)
				- *P*. *falcip*. *+ P*. *malariae* (4.9%)
				*- P*. *falcip*. *+ P*. *ovale* (0.6%)
2014	Matangila J.R. et al.	Healthy pregnant women in Kinshasa, the capital city of the DRC	29.5%	The study aimed to diagnose only asymptomatic *P*. *falciparum* infection
2016	Mvumbi D.M. et al.	Asymptomatic individuals randomly selected within 6 provinces of the DRC	48.2%	*- P*. *falciparum* (97.8%)
				- *P*. *falcip*.*+ P*. *malariae* (2.2%)
2020	This study	Adults aged at least 15 years old symptomatic or not, from all provinces of the DRC and recruited between October 2014 to March 2015	31%	*- P*. *falciparum* (96.3%)
				- *P*. *falcip*.*+ P*. *malariae* (3.7%)

Although comparable results were obtained, these studies were implemented in different settings. Indeed, the study of Taylor et al. [19] occurred at the national level during the Demographic and Health Survey in 2007 (Table 5). However, this study enrolled adults aged between 15 and 59 years. Matangila *et al*. focused their study on pregnant women infected with *P*. *falciparum* in Kinshasa, the capital city of the DRC [25] (Table 5). The study by Mvumbi *et al*. involved asymptomatic individuals recruited within only 6 provinces of the DRC [26] (Table 5). Our study involved all the provinces of the country and did not consider an age limit for adults. The extremes of age were 15 and 93 years. Thus, we included age categories that were not considered in previous studies.

We observed the highest prevalence of *Plasmodium* spp. in the province of Kasai Occidental, with 39.4%. This results are consistent with those of DHS-DRC II, which reported a high prevalence of malaria in some provinces, including Kasai Occidental [15]. The disparity in the *Plasmodium* spp. prevalence between provinces can be attributed to the differences in the application of measures for malaria control in these provinces. Indeed, consistent with a prior study [15], we observed that Kasai Occidental and Province Orientale are among the provinces with the lowest percentage of households possessing and using LLINs.

Similar to previous studies [27–29], we observed high parasite density in our study population. This can be explained by the fact that the DRC is located in areas exposed to equatorial and tropical epidemiological facies of malaria in which the transmission of *Plasmodium* is endemic [15].

We observed that women had a higher risk of being infected than men. This higher risk of *Plasmodium* infection can be related to the fact that women constitute approximately 60% of our study population, reflecting the current gender ratio of the country. In addition, this result can be explained by the fact that we include pregnant women, who, with children under five years of age, are most severely affected in Africa [30]. Previous studies have shown that, with a decrease in their immunity, pregnant women are at risk for *Plasmodium* infection [11, 12].

This study has some limitations. We did not consider pregnant women as a separate population from the women, and this could have affected the prevalence of *Plasmodium* infection in women. We could not directly assess the epidemiology of symptomatic malaria in the DRC, as our surveys did not include health facilities. Additionally, several of the variables included in the study were obtained from self-reports, which are subject to recall bias. Finally, as a cross-sectional survey, our study did not assess the effects of seasonality.

However, adult household surveys of malaria, at the national level, are not frequent in the DRC. Therefore, this study represents a contribution to the epidemiology of *Plasmodium* infections in adults (symptomatic or not) in the DRC. The use of molecular analysis strengthens the results of this study, as PCR assays are currently recognized as the most sensitive methods for the diagnosis and identification of *Plasmodium* [22, 26]. Furthermore, this study demonstrated elevated *Plasmodium* spp. infection and high parasite density in this age group. This high prevalence among adults may be related to the lack of access to prevention and treatment, such as insecticide-treated nets. These groups have not traditionally been the focus of intensive detection and control strategies. A previous study showed that while mothers slept with their young children under nets, other adults used bed nets less frequently [31]. In addition, entomological factors such as the predominance of outdoor biting vectors may contribute to the increased prevalence of *Plasmodium* spp. in adults [32]. Indeed, this age group spends more time outdoors than young children.

*Plasmodium* spp.-infected adults can play a critical role as transmission reservoirs. Adults are often asymptomatic or minimally symptomatic as many of them have acquired malarial immunity. Thus, the majority of *Plasmodium*-infected people are asymptomatic [33], and generally have low parasite density, which could be missed during parasite detection, thus perpetuating transmission [34].

Given their contribution to the pool of asymptomatic reservoirs of *Plasmodium* spp. infection and potentiality of transmission, targeting malaria interventions towards adults could maximize their impact.

## 5. Conclusion

The DRC, the second-most endemic country worldwide, has established a national policy for the fight against malaria. Strategic plans have been developed at the national level, and most of them are targeting at-risk groups (children under 5-years-old and pregnant women). Our study has shown that, despite all the efforts made in malaria control, the prevalence and parasite density of *Plasmodium* infection remain very high in adults. In addition, PCR analysis revealed that the use of microscopic assays underestimates the prevalence rate. Therefore, adults infected (symptomatic or asymptomatic) represent a reservoir for parasite transmission. Efforts toward malaria control and eradication should also target adults as the elimination of the parasites in only the at-risk groups (children and pregnant women) or only symptomatic patients, will not be effective as adult carriers will continue to act as parasite reservoirs.

## Supporting information

S1 Fig Sampling technique. A probabilistic sampling with four levels was carried out as sampling technique.
(DOCX)

S2 Fig Flowchart of samples included in analysis and data collection. (DOCX)

S1 Table Malaria prevalence in the DRC in adults by gender. (DOCX)

S2 Table Prevalence of malaria in adults by province using microscopy. (DOCX)

S3 Table Prevalence of malaria in adults by province by PCR. (DOCX)

S1 Questionnaire (PDF)

S2 Questionnaire (PDF)
