# Supplementary material for: Molecular detection and species identification of Plasmodium spp. infection in adults in the Democratic Republic of Congo: A population-based study
Source: PLoS One. 2020 Nov 23;15(11):e0242713. doi: 10.1371/journal.pone.0242713 (PMC7682816; doi:10.1371/journal.pone.0242713)
Supplement: S1 Fig — A probabilistic sampling with four levels was carried out as sampling technique. (DOCX) [file pone.0242713.s001.docx]

**
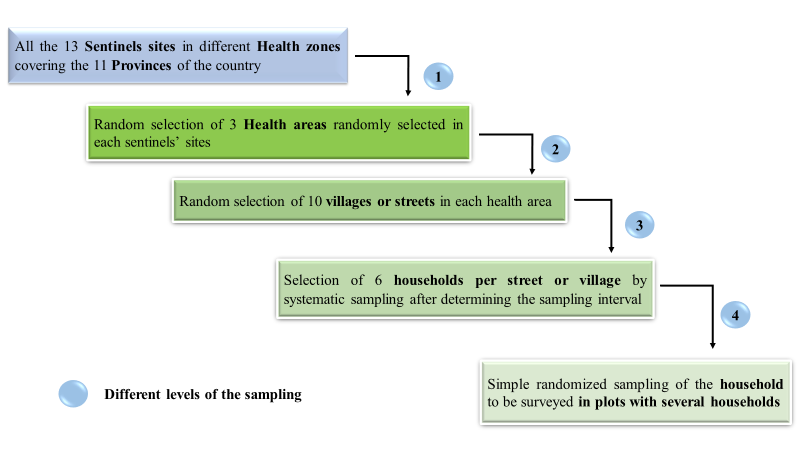
**

**S1 Fig. Sampling technique.** A probabilistic sampling with four levels was carried out as sampling technique.
