# Supplementary figures and images for: Molecular detection and species identification of Plasmodium spp. infection in adults in the Democratic Republic of Congo: A population-based study
Source: PLoS One. 2020 Nov 23;15(11):e0242713. doi: 10.1371/journal.pone.0242713 (PMC7682816; doi:10.1371/journal.pone.0242713)

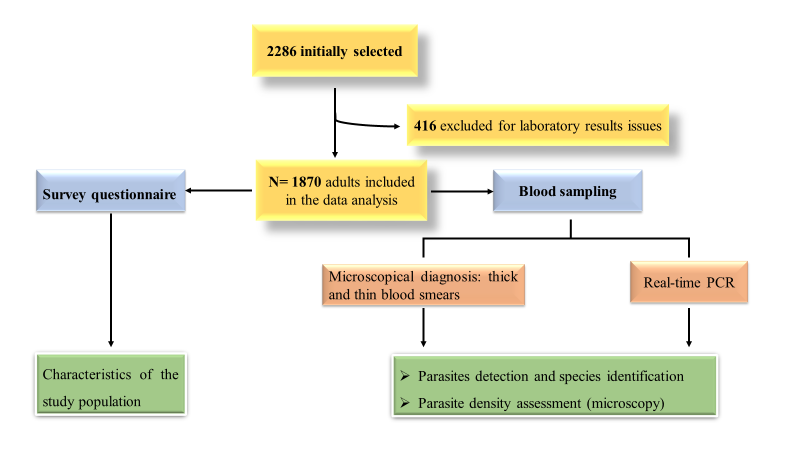
**S2 Fig. Flowchart of samples included in analysis and data collection**

Supplement: S2 Fig — (DOCX) [file pone.0242713.s002.docx]
