# Supplementary material for: Molecular detection and species identification of Plasmodium spp. infection in adults in the Democratic Republic of Congo: A population-based study
Source: PLoS One. 2020 Nov 23;15(11):e0242713. doi: 10.1371/journal.pone.0242713 (PMC7682816; doi:10.1371/journal.pone.0242713)
Supplement: S1 Table — (DOCX) [file pone.0242713.s003.docx]

**S1 Table. Malaria prevalence in the DRC in adults by gender**

| **Microscopy** | Negative N (%) | Positive N (%) | Total |
| --- | --- | --- | --- |
| **Gender** |  |  |  |
| Male | 614 (32.8) | 123 (6.6) | 737 |
| Female | 901 (48.2) | 232 (12.4) | 1133 |
| **Total** | **1515 (81)** | **355 (19)** | **1870** |
| **PCR** | Negative N (%) | Positive N (%) |  |
| **Gender** |  |  |  |
| Male | 479 (25.6) | 258 (13.8) | 737 |
| Female | 811 (43.4) | 322 (17.2) | 1133 |
| **Total** | **1290 (69)** | **580 (31)** | **1870** |
