# Supplementary material for: Molecular detection and species identification of Plasmodium spp. infection in adults in the Democratic Republic of Congo: A population-based study
Source: PLoS One. 2020 Nov 23;15(11):e0242713. doi: 10.1371/journal.pone.0242713 (PMC7682816; doi:10.1371/journal.pone.0242713)
Supplement: S2 Table — (DOCX) [file pone.0242713.s004.docx]

**S2 Table.** **Prevalence of malaria in adults by province using Microscopy**

| **Provinces** | **Microscopy** | **N** | | **%** | **95% CI** |
| --- | --- | --- | --- | --- | --- |
| Bandundu | positive | 27 |  | 15.6 | 11 to 22.5 |
|  | negative | 146 |  | 84.4 | 77.5 to 89 |
| Bas Congo | positive | 18 |  | 23.1 | 14.3 to 34 |
|  | negative | 60 |  | 76.9 | 66 to 85.7 |
| **Kasai Occidental** | **positive** | **45** |  | **30.6** | **23.9 to 39.5** |
|  | negative | 102 |  | 69.4 | 60.5 to 76.1 |
| Kasai Oriental | positive | 22 |  | 22.2 | 15.3 to 32.8 |
|  | negative | 77 |  | 76.8 | 67.2 to 84.7 |
| Katanga | positive | 80 |  | 23.5 | 19.2 to 28.5 |
|  | negative | 260 |  | 76.5 | 71.6 to 80.9 |
| Kinshasa | positive | 18 |  | 6.7 | 4 to 10.4 |
|  | negative | 250 |  | 93.3 | 89.6 to 96 |
| Maniema | positive | 63 |  | 18.0 | 15.5 to 24 |
|  | negative | 287 |  | 82.0 | 76 to 84.6 |
| Nord Kivu | positive | 11 |  | 9.1 | 4.6 to 15.7 |
|  | negative | 110 |  | 90.9 | 84.3 to 95.4 |
| Sud Kivu | positive | 30 |  | 24.0 | 19.6 to 35.9 |
|  | negative | 95 |  | 75.8 | 64.1 to 80.4 |
| Province Orientale | positive | 29 |  | 17.2 | 11.8 to 23.7 |
|  | negative | 140 |  | 82.8 | 76.3 to 88.2 |
