# Supplementary material for: Molecular detection and species identification of Plasmodium spp. infection in adults in the Democratic Republic of Congo: A population-based study
Source: PLoS One. 2020 Nov 23;15(11):e0242713. doi: 10.1371/journal.pone.0242713 (PMC7682816; doi:10.1371/journal.pone.0242713)
Supplement: S3 Table — (DOCX) [file pone.0242713.s005.docx]

**S3 Table. Prevalence of malaria in adults by province by PCR**

| **Provinces** | **PCR** | **N** | | **%** |
| --- | --- | --- | --- | --- |
| Bandundu | positive | 49/173 |  | 28.3 |
| Bas Congo | positive | 25/78 |  | 32.1 |
|  |  |  |  |  |
| **Kasai Occidental** | **positive** | **58/147** |  | **39.4** |
|  |  |  |  |  |
| Kasai Oriental | positive | 29/99 |  | 29.3 |
|  |  |  |  |  |
| Katanga | positive | 113/ 340 |  | 33.2 |
|  |  |  |  |  |
| Kinshasa | positive | 71 / 268 |  | **26.5** |
|  |  |  |  |  |
| Maniema | positive | 100/350 |  | 28.6 |
|  |  |  |  |  |
| Nord Kivu | positive | 28/121 |  | 23.1 |
|  |  |  |  |  |
| **Sud Kivu** | **positive** | **44/125** |  | **35.2** |
|  |  |  |  |  |
| **Province Orientale** | **positive** | **58/169** |  | **34.3** |
|  |  |  |  |  |
