# Supplementary material for: Molecular detection and species identification of Plasmodium spp. infection in adults in the Democratic Republic of Congo: A population-based study
Source: PLoS One. 2020 Nov 23;15(11):e0242713. doi: 10.1371/journal.pone.0242713 (PMC7682816; doi:10.1371/journal.pone.0242713)
Supplement: S1 Questionnaire — (PDF) [file pone.0242713.s006.pdf]

QUESTIONNAIRE

QUESTION. NUM. : ……………………………………………………………………………..

CODE RESPONDENT : ……………………………………………………………………………..

NAME : ……………………………………………………………………………..

AGE : ……………………………………………………………………………..

GENDER : ☐ M ☐ F

PROVINCE : ……………………………………………………………………………..

DISTRICT : ……………………………………………………………………………..

HEATH ZONE : ……………………………………………………………………………..

HEALTH AERA ……………………………………………………………………………

TELEPHONE : ……………………………………………………………………………..

ADRESSE : ……………………………………………………………………………..

EMPLOYMENT : ……………………………………………………………………………..

SCOOLING : ☐ YES ☐ NO

EDUCATION CAT. : ☐ NEVER ATTEND ☐ UNCOMPLETED PRIMARY SCHOOL
☐ COMPLETED PRIMARY ☐ UNCOMPLETED SECONDARY SCHOOL
☐ COMPLETED SECONADAIRY ☐ POST-SECONDARY
☐ PROFESSIONAL TRAINING

MARITAL STATUS : ☐ MARRIED ☐ SINGLE ☐ WIDOW
☐ COHABITATION ☐ DIVORCEE

NBR PERSONS/HOUSE: …………. NBR ROOMS: ……… NBR BEDS: ……...

POSSESSION LLIN : ☐ YES ☐ NO NBR LLIN : …………
USE OF LLIN : ☐ YES ☐ NO
INFO LLIN : ☐ YES ☐ NO ☐
LOCATION INFO LLIN : ☐ HOSP ☐ CARE SITES ☐ COMMUNITY
☐ CHURCH ☐ NGO
LASTING OF MOSQUITO NETS: ……………………………………………………….

BRANDS OF NETS : ☐ BRANDA ☐ BRAND B ☐ BRAND C
☐ PERMANET ☐ RONDE ☐ SERENA
☐ MANCHE ☐ BLEUE ☐ YOR KOOL
QUALITY OF NETS : ☐ WITH HOLES ☐ NO HOLES ☐ DID NOT CARE

HEALTH CONDITION : ☐ GOOD ☐ NOT GOOD

THICK SMEARS : ☐YES ☐NO

THIN SMEARS : ☐YES ☐NO

PCR ANALYSIS : ☐YES ☐NO

RESULTS TEST/PALU : ☐ YES ☐NO

POSITIF RESULTS : ☐YES ☐ NO

TREATED : ☐YES ☐ NO

DATE OF THE INTERVIEW: ………………………………………………………….

ABBREVIATIONS:

- QUESTION. NUM.: QUESTIONNAIRE NUMBER
- EDUCATION CAT.: EDUCATION CATEGORIES
- NBR PERSONS/HOUSE: NUMBER OF PERSONS IN THE HOUSEHOLD
- NBR ROOMS: NUMBER OF ROOM IN THE HOUSEHOLD
- NBR BEDS: NUMBER OF BEDS IN THE HOUSEHOLD
- LLINS : LONG-LASTING INSECTICIDAL MOSQUITO NETS
- LLIN INFO: INFORMATION ON THE LONG-ACTING INSECTICIDE-TREATED BED NET
- LOCATION INFO LLIN : THE PLACE WHERE THE RESPONDENT RECEIVED THE INFORMATION ON THE MOSQUITO NET
- HOSP : HOSPITAL
- NGO : NON-GOVERNMENTAL ORGANIZATION
- PALU : PALUDISME
- MED.CONSULTATION: MEDICAL CONSULTATION
