# Supplementary material for: Molecular detection and species identification of Plasmodium spp. infection in adults in the Democratic Republic of Congo: A population-based study
Source: PLoS One. 2020 Nov 23;15(11):e0242713. doi: 10.1371/journal.pone.0242713 (PMC7682816; doi:10.1371/journal.pone.0242713)
Supplement: S2 Questionnaire — (PDF) [file pone.0242713.s007.pdf]

FICHE D'ENQUETE

NUM QUESTION : ……………………………………………………………………………..

CODE PERSONNE : ……………………………………………………………………………..

NOMS : ……………………………………………………………………………..

AGE : ……………………………………………………………………………..

SEXE : ☐ M ☐ F

PROVINCE : ……………………………………………………………………………..

DISTRICT : ……………………………………………………………………………..

ZONE DE SANTE : ……………………………………………………………………………..

AIRESANTE :……………………………………………………………………………

TELEPHONE : ……………………………………………………………………………..

ADRESSE : ……………………………………………………………………………..
FONCTION : ……………………………………………………………………………..

SCOLARISE : ☐ OUI ☐ NON

NIVEAU SCOLAIRE : ☐ N'EST JAMAIS ALLEE ☐ PRIMAIRE PAS COMPLETE
☐ PRIMAIRE COMPLETE ☐ SECONDAIRE INCOMPLETE
☐ SECONADAIRE COMPLETE ☐ POST-SECONDAIRE
☐ FORMATION PROFESSIONNELLE

STATUTMATR : ☐ MARIE ☐ CELIBATAIRE ☐ VEUVE
☐ COHABITATION ☐ DIVORCEE

NBRE PERSONNES : ………… NBRE PIECES ……… NBRE LITS …………..

POSSESSIONMOUSTI : ☐ OUI ☐ NON NBRE MOUSTI : ………..
MOUSTI IMPREGNE : ☐ OUI ☐ NON
INFO MILD : ☐ OUI ☐ NON ☐ NE SAIT PAS
LIEU INFOMILD : ☐ CS/HOP ☐ SITES DES SOINS ☐ RECO/LEADERS
☐ EGLISE ☐ ONG
DUREE DU MOUSTI : ………………………………………………………
MARQUE MOUSTI : ☐ MARQUE A ☐ MARQUE B ☐ MARQUE C
☐ PERMANET ☐ RONDE ☐ SERENA
☐ BMANCHE ☐ BLEUE ☐ YOR KOOL
ETAT MOUSTI : ☐ AVEC DES TROUS ☐ SANS TROUS ☐ PAS OBSERVE

ETAT DE SANTE : ☐ BON ☐ PAS BON

GE : ☐ YES ☐ NO
FM : ☐ YES ☐ NO

PCR : ☐ YES ☐ NO

RESULTS TEST/PALU : ☐ YES ☐ NO

RESULTS POSITIF : ☐ YES ☐ NO

TRAITEMENT RECU : ☐ YES ☐ NO

DATE D'ENTRETIEN : …………………………………………………….

ABREVIATIONS :

NUMQUESTION : Numéro du questionnaire
HEURE ENQUE : Heure de l'enquête
CODE PERSONNE : Code de la personne enquêtée
GE : Goutte épaisse
FM : Frottis mince
STATUTMATR : Statut matrimonial
NBRE : Nombre
POSSESSIONMOUSTI : Possession d'une moustiquaire
MOUSTI : Moustiquaire
MILD ; Moustiquaire Imprégné d'insecticide a longue durée d'action
INFO MILD : Information sur la moustiquaire Imprégné d'insecticide a longue durée d'action
LIEU INFOMILD : Le lieu ou l enquêté a reçu les informations sur la moustiquaire
PALU : Paludisme
CONSULT : Consultation
RESULT : Résultat
MEDI : Médicament
INFO : Information
CS/HOP : Complexe scolaire/Hopital
RECO : Relais communautaire
ONG : Organisation non gouvernementale
